# Artificial intelligence applications in social media for depression screening: A systematic review protocol for content validity processes

**DOI:** 10.1371/journal.pone.0259499

**Published:** 2021-11-08

**Authors:** Priscilla N. Owusu, Ulrich Reininghaus, Georgia Koppe, Irene Dankwa-Mullan, Till Bärnighausen

**Affiliations:** 1 Institute of Global Health, University Hospital Heidelberg, Heidelberg, Germany; 2 Central Institute of Mental Health, Medical Faculty Mannheim, Heidelberg University, Heidelberg, Germany; 3 IBM Watson Health, Maryland, Bethesda, MD, United States of America; 4 Department of Global Health and Population, Harvard T.H. Chan School of Public Health, Boston, MA, United States of America; Erasmus Medical Center, NETHERLANDS

## Abstract

**Background:**

The popularization of social media has led to the coalescing of user groups around mental health conditions; in particular, depression. Social media offers a rich environment for contextualizing and predicting users’ self-reported burden of depression. Modern artificial intelligence (AI) methods are commonly employed in analyzing user-generated sentiment on social media. In the forthcoming systematic review, we will examine the content validity of these computer-based health surveillance models with respect to standard diagnostic frameworks. Drawing from a clinical perspective, we will attempt to establish a normative judgment about the strengths of these modern AI applications in the detection of depression.

**Methods:**

We will perform a systematic review of English and German language publications from 2010 to 2020 in PubMed, APA PsychInfo, Science Direct, EMBASE Psych, Google Scholar, and Web of Science. The inclusion criteria span cohort, case-control, cross-sectional studies, randomized controlled studies, in addition to reports on conference proceedings. The systematic review will exclude some gray source materials, specifically editorials, newspaper articles, and blog posts. Our primary outcome is self-reported depression, as expressed on social media. Secondary outcomes will be the types of AI methods used for social media depression screen, and the clinical validation procedures accompanying these methods. In a second step, we will utilize the evidence-strengthening **P**opulation, **I**ntervention, **C**omparison, **O**utcomes, **S**tudy type (**PICOS**) tool to refine our inclusion and exclusion criteria. Following the independent assessment of the evidence sources by two authors for the risk of bias, the data extraction process will culminate in a thematic synthesis of reviewed studies.

**Discussion:**

We present the protocol for a systematic review which will consider all existing literature from peer reviewed publication sources relevant to the primary and secondary outcomes. The completed review will discuss depression as a self-reported health outcome in social media material. We will examine the computational methods, including AI and machine learning techniques which are commonly used for online depression surveillance. Furthermore, we will focus on standard clinical assessments, as indicating content validity, in the design of the algorithms. The methodological quality of the clinical construct of the algorithms will be evaluated with the COnsensus-based Standards for the selection of health status Measurement Instruments (COSMIN) framework. We conclude the study with a normative judgment about the current application of AI to screen for depression on social media.

**Systematic review registration:**

International Prospective Register of Systematic Reviews PROSPERO (registration number CRD42020187874).

## Background

### Worldwide phenomenon of depression

Globally, depression is one of the leading causes of disability burdens affecting almost 300 million of the world’s population, with growing prevalence among children and adolescents [1, 2]. When left untreated, severe depression increases the risk of suicide, claiming 800,000 lives yearly, counting as the second leading mortality cause among 15–29 year-olds [1]. As many nations remain severely impacted by the SARS-CoV-2 pandemic, both clinical and subsyndromal levels of depression are expected to increase by unprecedented proportions [2–4]. Depression is associated with a complex interplay of biological, emotional, and social impairments on the affected individual [5]. Biologically, chronic exposure to depression-induced neurotransmitters is suspected to induce epigenetic changes which can alter brain functioning in the long term [6]. Kupferberg and colleagues (2016) [5] summarize the interpersonal deficits of depression into a framework with the subconstructs of “Affiliation and Attachment”, “Social Communication”, “Perception and Understanding of Self” and “Perception and Understanding of Others.” In addition, the economic impact of depression manifests as reduced work productivity and unemployment, contributing to a cumulative loss of $1 trillion each year to the global economy [7].

Despite the increasing global burden of depression, it remains underdiagnosed and poorly managed [8]. Literature evidence indicates that the multidimensional barriers to adequate care and improved outcomes for depression center around individual-level challenges including resistance to care-seeking and psychosocial limitations. At the clinical-level, diagnostic and treatment incongruity involving physician-oriented interventions are known barriers, while reduced access to formal mental health care services constitute structural-level problems linked to unmet care [9].

### Internet-based surveillance of depression

Internet-based sources including search queries, blogs, web encyclopedias, and social media websites provide proxy indicators for public health surveillance. This practice, termed “infoveillance” or “infodemiology,” aims to understand health trends, outbreaks, and disease prevalence from semi-structured data reported by internet users [10]. Social media networks such as Twitter, Facebook, TikTok, Reddit, Instagram, and blog sites each offer a virtual community network where people of various demographic backgrounds share sentiments, exchange information, and provide mutual support for common disease conditions. Remarkably, these platforms serve as channels for members of stigmatized populations such LGBTQ+ communities, and ethnic and religious minorities to coalesce anonymously [11].

Numerous studies have demonstrated the link between social media material and mental health status, including depression [12–15]. The surveillance of online content and users’ posting activity has been proposed as a complementary or even alternative precision tool for the early detection of depression markers. The strong appeal toward the analysis of social media content, as a form of patient self-reporting, arises from its potential as a measurement modality which is unconstrained by recall bias or selective disclosure. Moreover, risk factors including patient behavior, demographics, and socioeconomic status can be directly deduced from publicly accessible user profiles [16].

Artificial intelligence (AI) is a term that is broadly applied to the computerized automation of tasks normally requiring human intelligence [17, 18]. Through a subset of AI, known as machine learning (ML), algorithms or computer-generated operations can be developed to train large quantities of data obtained from social media postings to detect the presence of depression. With supervised learning ML methods, these algorithms gain the ability to recognize psychometric outcomes based on the degree of matching with the predefined indicator variables. For example, Coppersmith et al. (2015) [19] used linguistic analyses to predict depression and PTSD as well as demographic identities of Twitter users with age- and gender-matched controls. Yazdavar et al. (2020) [20] have similarly designed a multimodal framework to process visual imagery, textual data, and user interactions to detect depression among Twitter users. Further advanced applications of AI such as deep neural networks enable the matching of depressed individuals following in-depth evaluations of images based on characteristics of “colorfulness, facial presentation, sharpness, and naturalness.”

### Addressing the content validity of computational diagnoses for depression

Diagnostic manuals including the *Diagnostic and Statistical Manual of Mental Disorders*, fifth edition (DSM-V) and *International Classification of Disease* version 11 (ICD-11) are considered the gold standard for clinical practice. Following the administration of standardized psychiatric interviews, these frameworks provide a “behaviorally descriptive and systematic diagnostic system” [21] for identifying mental disorders, including depression. The psychopathology of a patient is determined by following a dimensionalized approach [21, 22] where a diagnosis is made on the basis of the degree of matching between his, her or their self-reported symptoms and the criteria description in the DSM-V manual. This “classical view” of diagnostic decision-making is predicated on strict adherence to the defined categories.

Even so, the DSM system is not without its unique set of limitations [23]. Conventionally, clinicians adopt a “probabilistic” approach to diagnosis, meaning that the most likely diagnosis is offered following the consideration of information relevant to a patient’s emotional state [22]. Given that a similar empirical methodology is used in computational psychiatry, there is a potential convergence point for the two disciplines to develop a modern, sophisticated standard for diagnosing mental disorders.

While previous studies [24–27] discuss the computational prediction of self-reported depression and other mental health disorders on social media, a remaining gap in the literature is the evidence of the scope of their clinical validity. Indeed, in the domain of psychiatry, Jablensky [28] notes that the nosology of neurological and psychological conditions remains unresolved. A valid diagnosis, however, is conceptualized as the level of correlation to the “idealized” norm, pathology, demographic and cultural variation of a reference population [28–30].

In their critical review of computational predictive techniques to determine mental health status on social media, Chancellor and De Choudhury [31] delve into the frameworks and practices underlying the working definition of a mental condition. Their review examines the methodology involved in such computational approaches, including the social media data sources, in addition to the selection of key variables, including linguistic style, topic content, and status disclosure. Of critical note is the question of ground truth approaches for establishing construct validity, *i*.*e*. a thorough enquiry into the “theoretical construct of knowledge [pertaining] to the observed phenomenon [of mental health status] within the dataset” [31]. The authors expound on the potential value of “digital psychiatry” in complementing the traditional clinical approaches to mental health treatment; meanwhile, admonishing that “stronger connections to traditional psychiatry” are essential for the purpose of accurate diagnoses.

Whereas Chancellor and De Choudhury give an overview of the practice of using automated predictive techniques on social media to determine mental health status, in addition to the construct validity of these techniques, our forthcoming systematic review will analyze content validity, with respect to the contribution of clinical diagnostic frameworks in computational methods used to identify depression on social media. Of vital note, we consider social media content on depression as a *self-reported* health status indicator. Through the lens of the COSMIN framework [32], we aim to critique the performance of these computational instruments in measuring the clinical construct of depression. Likewise, we will pose a normative judgment about the observed practices in the use of predictive techniques on social media for self-reported depression against current clinical diagnostic norms.

## Methods

### Objectives

**Fig 1:**
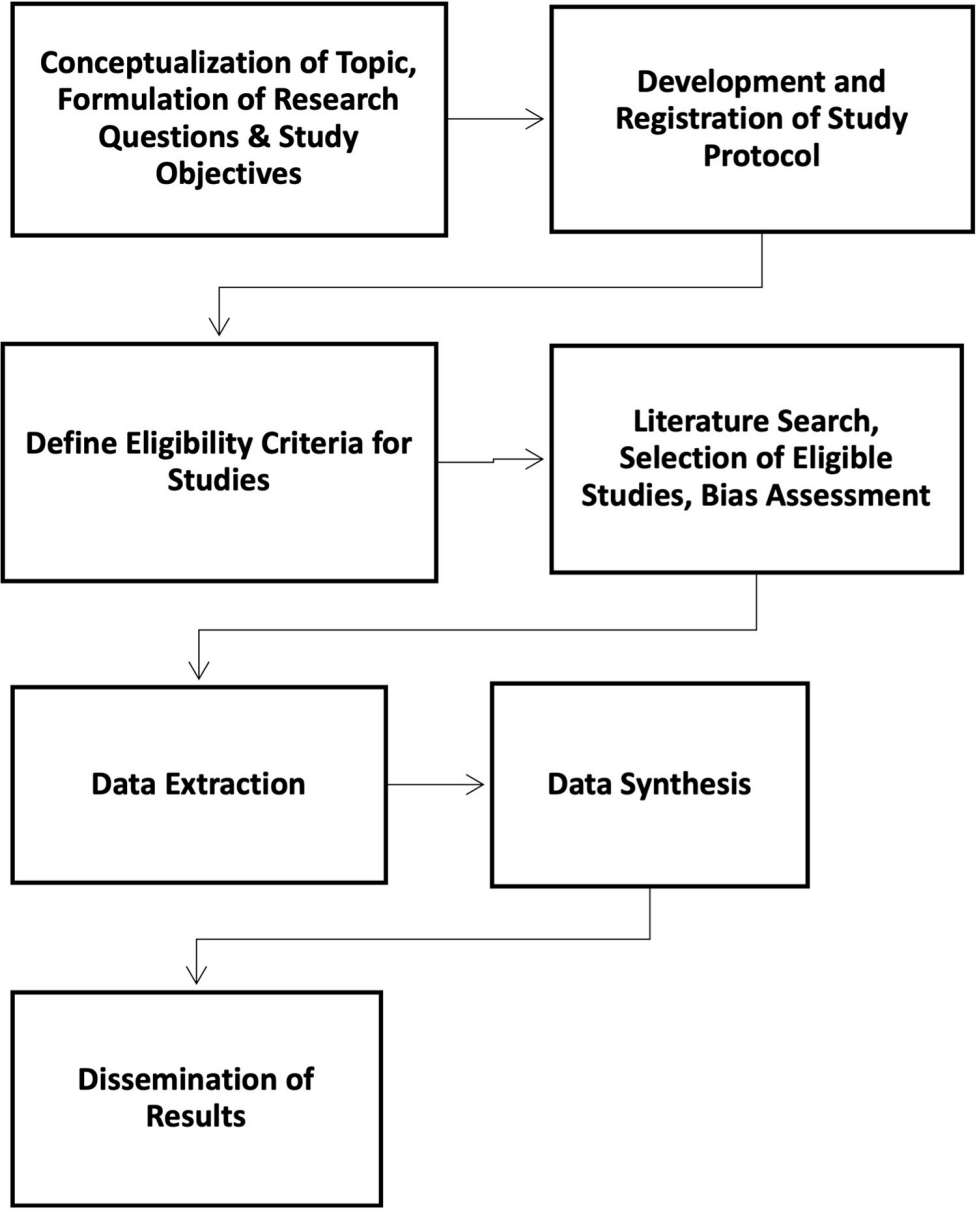


The purpose of this impending systematic review is to present the structures and processes employed in the design of AI methods including ML algorithms for detecting depression on social media. Primarily, this research will focus on whether clinical depression screening instruments are integrated into the design of algorithms for diagnostic assessments of social media user-generated content. In this study, social media content depression will be considered as patient self-reported health outcomes. Using the COSMIN checklist [32], we will appraise the measurement properties of the computational methods with respect to content validity against clinical diagnostic instruments. A flowchart summary of the steps to be followed in the preparation of this systematic review is illustrated in Fig 1.

### Protocol and registration

The upcoming systematic review will be prepared according to the Preferred Reporting Items for Systematic Reviews and Meta-Analyses (PRISMA) [33] guidelines for evidence-based reporting. The review protocol is registered with the University of York’s online international prospective register of systematic reviews (PROSPERO; registration ID CRD42020187874). The authors will document any amendments to the protocol in the PROSPERO record.

### Eligibility criteria

#### Participants

The Pew Research Center [34] has studied the demographic profile of heavy users of social media. Per its findings, young people between the ages of 18 and 29 comprise the highest proportion of adults subscribed to at least one social media site. However, as depression is not only limited to this age demographic, the systematic review will consider reports about male, female and gender diverse social media users worldwide, of all ages. Any existing social media websites will be considered.

### Outcomes

**Primary outcome.**

- Self-reported depression on social media

**Secondary outcomes.**

- The wide-ranging application of AI in detecting depression on social media websites.
- Demographic profile of social media users screened for depression.
- Distinct subsets of AI methods employed.
- Structures and processes involved in the selection of eligible postings for depression screening.
- Clinical assessments and standards used in identifying depression cases on social media.

Our reviews will report on the absence or presence of clinical frameworks in the design of ML algorithms. Since the review focuses on the clinical diagnosis of depression, we will not consider studies that refer to the broad spectrum of mental health disorders.

### Types of studies

The impending systematic review will consider research publications discussing the use of AI and ML in the context of social media for the detection of treatment of depression. We will consider all existing literature from peer reviewed and some gray literature sources published from 2010 to present. Study designs will be inclusive of cross-sectional, cohort, and case-control studies, in addition to randomized controlled trials available in English or German languages. Publications from both high-income and developing country settings will be considered. Gray literature sources will be limited to conference reports. We will not consider newspaper, magazine articles, opinions/commentaries, dissertations, editorials, blog posts, and policy briefs. The number of excluded studies at each stage will be documented and shown in the review manuscript.

#### Information sources and search strategy

**Fig 2:**
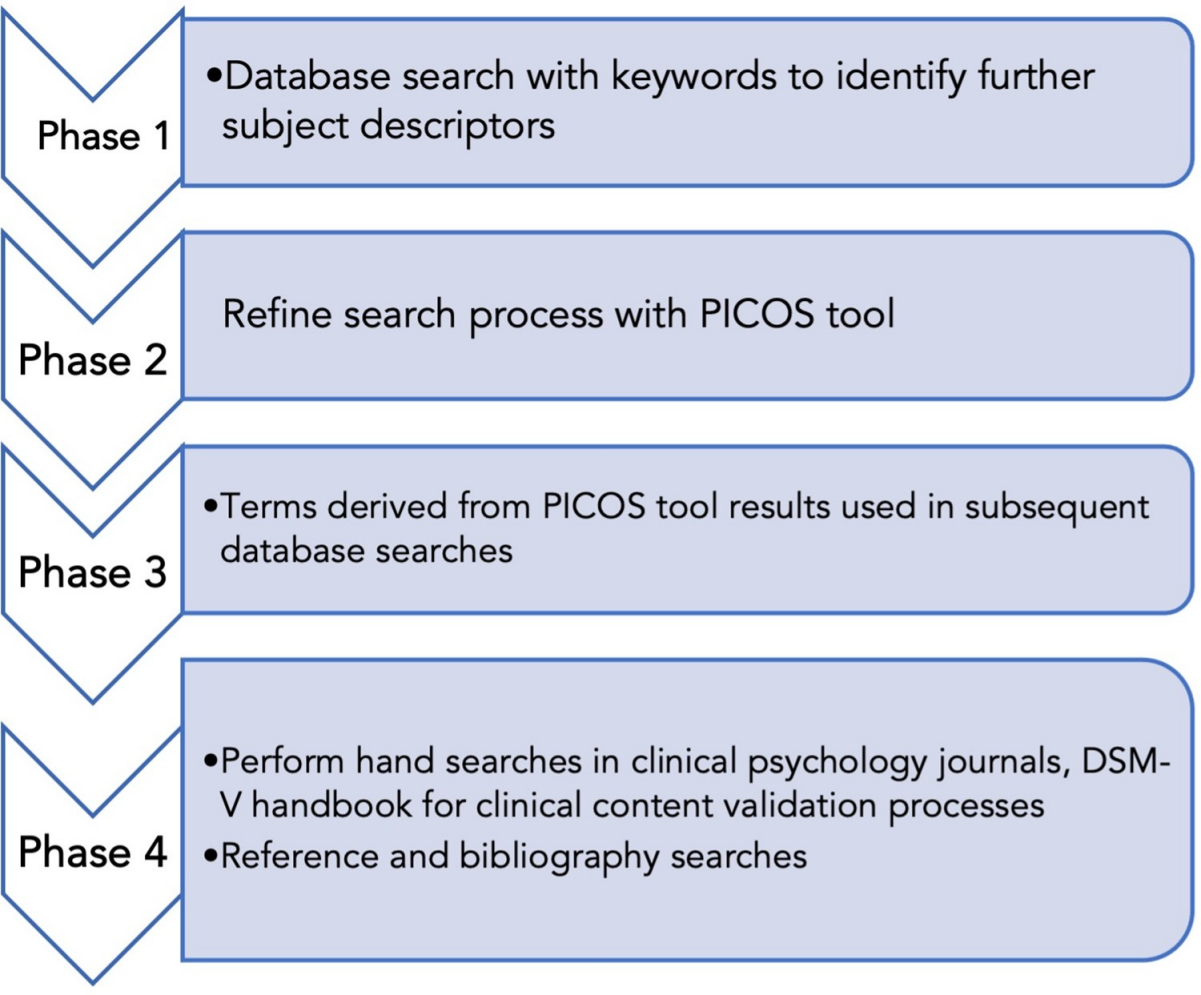
Key phases in literature searches.

English and German language studies from 2010 to 2020 meeting eligibility criteria will be searched from PubMed, APA PsychInfo, Science Direct, EMBASE Psych, Google Scholar, and Web of Science databases. The review will follow a rigorous search process from both scientific publications and selected gray literature sources. It will be performed in four phases as shown in Fig 2.

In phase 2 of the search process (Fig 2), the main keywords will be entered into the PICOS [35] tool available in PubMed, where **P**opulation is “social media users”; **I**ntervention is “artificial intelligence” or “machine learning” or “Natural Language Processing (NLP)” use for sentiment screening; **C**omparison is standard “psychiatric diagnostic tools” used in clinical settings; **O**utcomes are “depression,” “AI applications,” “structures and processes used in social media depression screenings,” and “clinically-derived standards applied in these processes”; **S**tudy design is “cross-sectional”, “case-control”, “case series”, “randomized controlled trial.”

Preliminary search keywords to be used are “Artificial intelligence”, “social media”, “depression”, “Artificial intelligence AND social media AND depression”, “Machine learning AND social media AND behavioral health”, “social media AND depression”, “Natural Language Processing (NLP) AND social media AND depression.”

#### Study selection

After applying search terms and keywords, all identified records from our databases will be exported to the Endnote® research information management system. The total number of records will be noted, with duplicates record numbers deducted from this amount. The selection of eligible studies will involve a preliminary skim through titles and abstracts, with non-relevant records eliminated. This process will be independently conducted by two reviewers on the authorship team, and final selections will be compared. A third reviewer will be consulted to reconcile any disagreements with study selections. Selection processes and tallies will be documented in the PRISMA flow chart.

### Data management and extraction

The data extraction process will be facilitated by the Systematic Review Data Repository (SRDR) web interface offered by the U.S. Department of Health and Human Services Agency for Healthcare Research and Quality (AHRQ). This free online tool allows project managers to define the outcomes of the systematic review, and to import records with information pertaining to the outcomes. This will include demographic characteristics of target population, study type, settings, interventions and comparator studies. The SRDR tool has the capacity to produce graphical and statistical summaries of extracted data. Data extraction will be performed by two independent reviewers, and results discussed with a third reviewer, in case of divergencies.

### Risk of bias

Siemieniuk and Guyatt [36] describe bias as occurring when there is the failure to report the complete truth to a phenomenon, due “inherent limitations in the design or conduct of a study.” Therefore, to avoid the risk of bias, publications meeting the eligibility criteria for this systematic review will be evaluated through the Grading of Recommendations, Assessment, Development and Evaluations (GRADE) framework [37]. The GRADE is a widely used tool to rate the quality of evidence for the desired outcome to be presented in the review. At every stage of the review process, information regarding the reasons for exclusion will be documented. Expert opinion will be sought for publications whose eligibility warrants further debate.

### Patient and public involvement

This prospective study will not directly involve either patients or the general public. We will utilize secondary data reporting obtained from published peer review and selected gray literature sources.

### Data synthesis

For this report, evidence will be qualitatively synthesized according to themes. Thematic synthesis derives from the grounded theory method used to contextualize health-related data by overarching concepts [37]. Previous work describes this approach as involving iterative collection and analysis of data resulting in the emergence of theories from the evidence [38].

Where possible, meta-analyses will be conducted following considerations about selected publications’ study design, reported findings, and outcomes.

In evaluating the content validity of selected studies, we will apply the COSMIN checklist in its binary form for evaluating the properties of measurement instruments for patient-reported health outcomes. It will be accompanied by a four-item checklist (see supplementary information) for the type of evidence presented in the literature, with composite scores out of 20 assigned for each selected publication. Additional quantitative analyses will involve sensitivity analyses to determine robustness of our outcome variables.

## Discussion

Mental illness persists as a highly stigmatized condition. The double burden is especially pronounced among ethnic and sexual minority groups, whose self-stigmatization results in feelings of embarrassment, shame, and poor health seeking behavior [39]. Cross-cultural barriers in patient-provider communication also contribute to lower patient satisfaction, misdiagnoses, and poor retention of minorities in mental health care settings [40]. Social media functions as a valuable outlet for individuals with mental illness to find validation, hope and peer-to-peer support to cope with their condition [41, 42]. Digitalized records of user engagement in online peer networks contain rich insight into the peculiar burdens faced by people suffering from mental disability.

On social media, everyday language is effected in describing sentiments, as well as to chronicle life events. User characteristics including social connectedness, demographics, and socioeconomic status may be inferred from materials often voluntarily shared by individuals [42]. In some instances, users provide direct evidence of mental illness by self-reporting or through membership in support groups on social media such as subreddit’s r/depression, r/anxiety, r/ADHD, and various Facebook groups for mental health. Contemporary advances in big data analytics propose the analysis of language use and linguistic styles in social media content to predict ongoing or future incidence of mental illness. For example, De Choudhury and coworkers [43, 44] have that found that prior Facebook postings can serve as indicators of postpartum depression in new mothers. Computational algorithms have been trained to not only identify the presence of mental illness, but also to enable the multiclassification of psychiatric conditions with over 70% accuracy [44].

In 2001, a blueprint for effective depression care management was developed using the template of the Chronic Care Model by Wagner and colleagues [45–50]. This framework begins with the “systematic identification of patients at increased risk for depression,” and recommends the use of an assessment tool that allows patients to be categorized by “episode” as well as “severity.” Within the domain of psychometrics, validity, which is a property of instrument quality for measuring patient-reported health outcomes, is necessary to reduce biased estimates and false diagnoses [50]. Content validity focuses on the instrument’s accuracy in “representing” the clinical domain in question [30, 50]. From the available evidence, the instrument or algorithm performs qualitative or quantitative assessments of the indicators and formulates a conclusion in accordance with established clinical benchmarks. Thus, if AI applications are being implemented as alternative precision tools for depression screening, then the core requirements of this blueprint for effective depression care management can similarly be applied toward the systematic identification and stratification of high-risk individuals.

Our forthcoming systematic review aims to investigate the content validity of these computational methods, taken as the clinical structures and processes involved in identifying social media users exhibiting depression symptoms. We will assess the extent to which the depression screening conducted by newly developed, automated instruments reflect the variables measured in clinical practice. In capturing the parallels between these modern computational methods and gold standard clinical frameworks, we seek to establish the content validity of these AI and ML algorithm tools in social media-based depression screening.

### Study limitations

Psychiatric epidemiology [51, 52], comprising population-based studies of mental disorders, employs both self-reports and structured diagnostic interviews [53]. Within this practice, weak concordance between self-reported and standard clinical assessments is frequently observed [53]. In psychiatric practice, a known maxim is that a person’s own “claim of mental impairment is alone not enough….” [53–55]. As such, this tenet highlights the inherent limitation of online content, taken as self-reports, as a sufficient data source for population-level inferences of mental health status. Considering the need for objective medical examination to triangulate self-disclosed disability, this systematic review will overlook any observed discrepancies by focusing on the clinical frameworks utilized in validating depressive symptoms.

### Ethics, privacy, and dissemination

Ethics scholars deliberate on the dilemmas of privacy and user consent for research purposes in this emerging field of “social computing” [56]. Undeniably, this question falls into the wider theme of the societal impacts of AI in consumer products. As early as the 1980s and 1990s, ethics educators have highlighted the challenge for digital computing applications to responsibly protect marginalized communities from harm through the integration of good ethical practices [56, 57]. Ethical and privacy concerns are to be carefully resolved as social media users increasingly include minors who may not fully grasp the implication of digitally traceable personal information. For academics, we still confront the controversy surrounding the norms and best practices for collecting and disseminating public content for research purposes [58, 59].

This ongoing study is being prepared according to the guidelines of the PRISMA checklist. Information will be derived entirely from secondary source material in peer reviewed literature and selected gray sources, as aforementioned. The completed systematic review including the dataset of literature findings will be published and shared at conferences and proceedings related to clinical psychiatry, big data research, and artificial intelligence methods.

## Supporting information

S1 File Four-item checklist to evaluate systematic review literature evidence. (PDF)

S2 File PRISMA 2009 flow diagram. (DOC)

S3 File PRISMA-P checklist. (PDF)

## List of abbreviations

AHRQ: Agency for Healthcare Research and Quality
AI: Artificial Intelligence
COSMIN: Consensus-based Standards for the selection of health status Measurement Instruments
GRADE: Grading of Recommendations, Assessment, Development and Evaluations framework
LGBTQ+: Lesbian, gay, bisexual, transsexual, queer, and others
ML: Machine learning
PICOS: Problem, Intervention, Comparison, Outcomes, Study type
SRDR: Systematic Review of Data Repository
