# Supplementary material for: Artificial intelligence applications in social media for depression screening: A systematic review protocol for content validity processes
Source: PLoS One. 2021 Nov 8;16(11):e0259499. doi: 10.1371/journal.pone.0259499 (PMC8575242; doi:10.1371/journal.pone.0259499)
Supplement: S1 File — (PDF) [file pone.0259499.s001.pdf]

Supplementary information

Four-item pre-checklist for content validity in AI-based depression screening

**Item**	**Score**
1. Working definition of depression stated in the study?	Yes ☐ No ☐
2. Does definition fit current clinical standard? E.g. DSM-V, ICD-11?	Yes ☐ No ☐
3. Consultation with psychiatric or clinical experts?	Yes ☐ No ☐
4. Heterogeneity of results (Give brief description)	Yes ☐ No ☐
Total score Yes=1; No = 0	

COSMIN Checklist for content validity

**Box D. Content validity (including face validity)**				
General requirements		**yes**	**no**	**?**
1	Was there an assessment of whether all items refer to relevant aspects of the construct to be measured?	☐	☐	☐
2	Was there an assessment of whether all items are relevant for the study population? (e.g. age, gender, disease characteristics, country, setting)	☐	☐	☐
3	Was there an assessment of whether all items are relevant for the purpose of the measurement instrument? (discriminative, evaluative, and/or predictive)	☐	☐	☐
4	Was there an assessment of whether all items together comprehensively reflect the construct to be measured?	☐	☐	☐
5	Were there any important flaws in the design or methods of the study?	☐	☐	

Source: Mokkink et al. 2010. Qual Life Res (2010) 19:539–549
